# Culture Medium for the Gonococcus

**Published:** 1914-01

**Authors:** 


CULTURE MEDIUM FOR THE GONOCOCCUS. Sabouraud and Noiré *Annales de derm. et syph.,* vol. iv, No. 7, July, 1913.

(1.) A litre of fresh milk is boiled for five minutes; (2) the casein is then precipitated with 2 c.cm. of hydrochloric acid, and the serum recovered by simple passage through a piece of linen; (3) the filtrate is added to half its quantity of water and the mixture neutralized with 10 per cent. soda solution; (4) it is then autoclaved at 120° for ten minutes; (5) the following are then added in the strength indicated: Peptone 1 in 100, glucose 1 in 100, urea o.3 in 100, agar 1.6 in 100; (6) filteration through filter paper and division into separate test-tubes, which are sterilized for ten minutes at 110° C., completes the preparation.

The gonococci are incubated in twenty-four hours.

